# Account of Diseases in an Eastern District of London, from the 20th of June to the 20th of July, 1801

**Published:** 1801-08

**Authors:** 


*Account of Diseases in an Eastern District of London, from the 20th of June to the 20th of July,* 1801.

| | No. of Cases. |
|---|---|
| ACUTE DISEASES. | |
| Typhus | 22 |
| Febris Intermittens | 1 |
| Pneumonia | 7 |
| Cynanche Tonsillaris | 8 |
| Acute Rheumatism | 3 |
| CHRONIC DISEASES. | |
| Cough | 18 |
| Dyspnœa | 9 |
| Cough and Dyspnœa | 12 |
| Phthisis Pulmonalis | 3 |
| Pleurodyne | 1 |
| Hydrothorax | 2 |
| Apoplexia | 1 |
| Paralysis | 2 |
| Cephalalgia | 5 |
| Dyspepsia | 7 |
| Anasarca | 4 |
| Ascites | 2 |
| Amenorrhœa | 3 |
| Menorrhagia | 2 |
| Chlorosis | 5 |
| Asthenia | 2 |
| Hypochondriasis | 2 |
| Hysteria | 3 |
| Scrophula | 2 |
| Diarrhœa | 5 |
| Hæmorrhois | 3 |
| Scabies | 1 |
| Herpes | 3 |
| Chronic Rheumatism | 12 |
| PUERPERAL DISEASES. | |
| Menorrhagia Lochialis | 3 |
| Low Puerperal Fever | 2 |
| Abscessus Mammarum | 1 |
| INFANTILE DISEASES. | |
| Febris Infantilis | 2 |
| Rachitis | 1 |
| Aphthæ | 5 |
| Herpes | 8 |

It will appear from the annexed list, that the fever, which has long formed a large proportion in former lists, continues to prevail, and that the number of patients under its influence is still large. This disease has propagated itself to a considerable extent, particularly amongst the lower orders of society; so that there is hardly a family that has been visited by it, in which almost every member of it has not been more or less affected. Together with the other symptoms of this disease, which have been frequently recited, some affections of the throat have of late, in several instances, been experienced. In these cases there has been a slight inflammation of the mucous membrane of the fauces, and some enlargement of the tonsils, occasioning a degree of pain and difficulty in deglutition. These symptoms have, however, soon yielded to the inhaling of the steam of warm water, or frequently sipping some tepid emollient liquor, or the use of moderately astringent gargles.

Similar affections of the throat, but in a higher degree, have in some instances constituted the primary disease. The patient has first complained of stiffness and fullness about the throat, with difficulty of deglutition; the tonsils and the whole of the internal fauces have been much inflamed, and the degree of fever indicated by the fullness and frequency of the pulse, and the heat of the skin, has been considerable.

The uſe of aperient medicines, keeping up a determination to the ſkin by antimonials, and the uſe of emollient gargles, have generally been attended with ſucceſs, and in a few days the diſeaſe has been removed.

